# Integration of RNA-Seq data with heterogeneous microarray data for breast cancer profiling

**DOI:** 10.1186/s12859-017-1925-0

**Published:** 2017-11-21

**Authors:** Daniel Castillo, Juan Manuel Gálvez, Luis Javier Herrera, Belén San Román, Fernando Rojas, Ignacio Rojas

**Affiliations:** Department of Computer Architecture and Technology, University of Granada, Periodista Rafael Gómez Montero, 2, Granada, 18014 Spain

**Keywords:** RNA-Seq, Microarray, Breast cancer, Cancer, SVM, Random Forest, k-NN, Gene expression, Classification, Integration

## Abstract

**Background:**

Nowadays, many public repositories containing large microarray gene expression datasets are available. However, the problem lies in the fact that microarray technology are less powerful and accurate than more recent Next Generation Sequencing technologies, such as RNA-Seq. In any case, information from microarrays is truthful and robust, thus it can be exploited through the integration of microarray data with RNA-Seq data. Additionally, information extraction and acquisition of large number of samples in RNA-Seq still entails very high costs in terms of time and computational resources.This paper proposes a new model to find the gene signature of breast cancer cell lines through the integration of heterogeneous data from different breast cancer datasets, obtained from microarray and RNA-Seq technologies. Consequently, data integration is expected to provide a more robust statistical significance to the results obtained. Finally, a classification method is proposed in order to test the robustness of the Differentially Expressed Genes when unseen data is presented for diagnosis.

**Results:**

The proposed data integration allows analyzing gene expression samples coming from different technologies. The most significant genes of the whole integrated data were obtained through the intersection of the three gene sets, corresponding to the identified expressed genes within the microarray data itself, within the RNA-Seq data itself, and within the integrated data from both technologies. This intersection reveals 98 possible technology-independent biomarkers. Two different heterogeneous datasets were distinguished for the classification tasks: a training dataset for gene expression identification and classifier validation, and a test dataset with unseen data for testing the classifier. Both of them achieved great classification accuracies, therefore confirming the validity of the obtained set of genes as possible biomarkers for breast cancer. Through a feature selection process, a final small subset made up by six genes was considered for breast cancer diagnosis.

**Conclusions:**

This work proposes a novel data integration stage in the traditional gene expression analysis pipeline through the combination of heterogeneous data from microarrays and RNA-Seq technologies. Available samples have been successfully classified using a subset of six genes obtained by a feature selection method. Consequently, a new classification and diagnosis tool was built and its performance was validated using previously unseen samples.

## Background

Cancer is the second leading cause of death worldwide, just behind cardiovascular disease. Specifically, breast cancer is one of the five most dangerous cancers in the world, showing a high mortality rate according to World Health Organization (WHO), and being the cancer with the highest impact among the female population [1]. Nowadays, many breast cancer diagnoses are performed when a patient presents several related symptoms, thus increasing the mortality risk. If the cancer has spread, treatment becomes more difficult, and generally the chances of surviving are significantly lower. However, cancers that are diagnosed at an early stage are more likely to be treated successfully. Therefore, it is primordial to find biomarkers that allow an early diagnosis of breast cancer. Two sequencing technologies, microarray and RNA-Seq, have been used for obtaining gene expression. They are briefly described next.

### Microarray technology

Microarray has been the main sequencing technology used in the last two decades until the arrival of Next Generation Sequencing techniques. The most extended microarray platforms are Affymetrix [2] and Illumina [3], leading the second one the RNA-Seq sequencing technology market. Nevertheless, there are other very important microarray manufacturers such as Agilent [4], Exiqon [5] or Taqman [6]. A high simultaneous number of genes can be measured at expression level from the use of microarrays. The expression values are achieved by means of microscopic DNA spots attached to a solid surface which have followed a hybridization process. Once this process is completed, it is possible to read the expression values with a laser, and consequently store the quantification levels in a.CEL file [7].

### RNA-Seq technology

As a natural evolutionary step in the treatment of biological information from DNA, RNA-Seq is gradually replacing the widespread use of microarrays. Although its application was originally intended for genomic transcription study, it also allows achieving a mapping between the levels of transcription and gene expression [8]. In this sense, its combination with other functional genomics methods allows enhancing the analysis of gene expression. This is achieved through the quantification of the total number of reads that are mapped to each locus in the transcriptome assembly step. RNA-Seq read counts robustness has been validated against predecessor technologies such as microarrays or quantitative polymerase chain reaction (qPCR) [9].

### Comparison between both technologies

RNA-Seq offers an important number of advantages over microarrays, although the cost of RNA-Seq experiments is also higher than in microarray technology nowadays:

- RNA-Seq allows detecting the variation of a single nucleotide.
- RNA-Seq does not require genomic sequence knowledgement.
- RNA-Seq provides quantitative expression levels.
- RNA-Seq provides isoform-level expression measurements.
- RNA-Seq offers a broader dynamic range than microarrays.

In spite of these advantages, microarrays are still used due to their lower costs. Besides, as microarrays have been used for a longer period, there exist many robust statistical and operational methods for their processing [10–15].

There are many significant microarray experiments already available to the research community, and there is also even a high number of microarray datasets that have not been analyzed so far. These datasets might have information that could reveal important facts and candidate biomarkers. In any case, there is no doubt that RNA-Seq is the present technology, but it can also take advantage of the available data from microarray technology. As Nookaew et al. explained [16], there is a high consistency between RNA-Seq and microarray, which encourages to continue using microarray as a versatile tool for gene expression analysis.

The main objective of this work is to find possible breast cancer biomarkers from patient and control samples acquired via NCBI GEO web platform [17]. To this end, an exhaustive search has been done in order to obtain statistically significant samples from both microarray and RNA-Seq series. Two datasets have been considered in this study, one for training and one for testing. The training dataset has been used to extract the Differentially Expressed Genes (DEGs), and to design a classifier. The test dataset has been considered for the assessment of the DEGs selection and classification processes.

In the case of RNA-Seq samples, cqn package [18] has been used to calculate the expression values from the BAM/SAM file. Once the expression values were available, they were merged and normalized with the microarray data. Gene expression was achieved through a joint study of all series that allowed integration among microarrays and RNA-Seq data.

Most of the previous studies in the selection of biomarkers perform this process through statistical tools over a given dataset and a given technology. However, this work takes an innovative step forward by combining different datasets and microarray technologies together with RNA-Seq data. Furthermore, this research also builds an smart breast cancer classifier with the aim of achieving early diagnosis when unlabeled samples are presented. To this end, the minimum-Redundancy Maximum-Relevance (mRMR) [19] feature selection algorithm was applied in order to select the most relevant genes to perform the classification. Also, three different classification algorithms have been implemented and their results compared. The first classifier makes use of Support Vector Machines (SVM) [20, 21]. Alternatively, Random Forest (RF) [22] and k-Nearest Neighbor (k-NN) [23] classifiers have also been designed.

This paper has been structured as follows. This section has shown the introduction and state of the art of this work. Next section explains the methodology followed in this study. It begins by describing the available data series that have been used for this research. Later, the pipeline for processing and classifying the data is shown. An innovative step for automatic sample classification is described using machine learning techniques. The results and discussion section shows the integrated gene expression, revealing those genes that remain unchanged regardless of the technology used in the gene expression identification process. Furthermore, this section underlines the validity of the proposed approach and its utility in breast cancer early diagnosis using the developed classification tool. Finally, the conclusions section summarizes the most important contributions of this study for breast cancer diagnosis and genetic profiling.

## Methods

### Microarray and RNA-Seq series

The first issue that must be addressed concerns the definition of the kind of sample that is going to be used, along with the determination of the tissue or cell that the sample comes from. As a result, a wide search through the NCBI-GEO platform has been done with the objective of finding datasets belonging to both the selected cell lines and the considered technologies. In this study, control samples have been selected from the MCF10A cell line [24]. This cell line is classified as a healthy non-tumorigenic epithelial cell line. Various breast cancer cell lines were selected as cancer samples (MCF7 and HS578T) [25, 26]. Besides, not every sample from each of the series has been selected, as there are samples that do not belong to the cell lines required, or they have been treated with some kind of drug that could produce some noise in the final results.

Once the requirements for selecting the desired samples were established, an exhaustive search of Affymetrix and Illumina series was carried out for microarray data. On the other hand, RNA-Seq data was selected from Illumina HiSeq technology. Only datasets containing the above-mentioned cell lines were selected. Table 1 summarizes the selected series for this study. As it can be seen, the NCBI GEO database offers a larger availability of microarray data when compared with the number of RNA-Seq samples. Two separated supersets have been created, one for training predictive models, and the other for their testing, both containing microarray as well as RNA-Seq samples. The training dataset is made up of 108 microarray samples: 65 samples from Affymetrix, 43 from Illumina, and 24 RNA-Seq samples. On the other hand, the test set is made up of 120 samples of microarray (108 of Illumina and 12 of Affymetrix) as well as 6 samples of RNA-Seq. These series are publicly available at https://www.ncbi.nlm.nih.gov/geo/query/acc.cgi?acc=S.NAME where S.NAME is the name of each series at NCBI GEO.

**Table 1:** Description of the training and test series considered with number of samples/outliers

TRAINING SERIES					
Series	Platform	Technology	Quality samples	Excluded outliers	Samples origin
GSE52712	Affymetrix	Microarray	19	1	Manchester (UK)
GSE40987	Affymetrix	Microarray	10	0	Boston (USA)
GSE52262	Affymetrix	Microarray	16	0	Houston (USA)
GSE12790	Affymetrix	Microarray	20	1	San Francisco (USA)
GSE46834	Illumina	Microarray	8	0	New York (USA)
GSE68651	Illumina	Microarray	35	1	Southampton (UK)
GSE74251	Illumina	RNA-Seq	12	0	Philadelphia (USA)
GSE74377	Illumina	RNA-Seq	12	0	Iowa (USA)
TOTAL	Integrated		132	3	
TEST SERIES					
Series	Platform	Technology	Quality samples	Excluded outliers	Samples origin
GSE78011	Illumina	RNA-Seq	3	0	Louisville (USA)
GSE81593	Illumina	RNA-Seq	3	0	New York (USA)
GSE75292	Illumina	Microarray	6	1	Goyang (South Korea)
GSE29327	Affymetrix	Microarray	6	0	South San Francisco (USA)
GSE30931	Illumina	Microarray	12	0	Goettingen (Germany)
GSE48398	Illumina	Microarray	36	0	Texas (USA)
GSE35928	Affymetrix	Microarray	6	0	Piscataway (USA)
GSE57339	Illumina	Microarray	12	0	New Haven (USA)
GSE45715	Illumina	Microarray	42	0	Miami (USA)
TOTAL	Integrated		126	1	

### Microarray pipeline

**Fig. 1:**
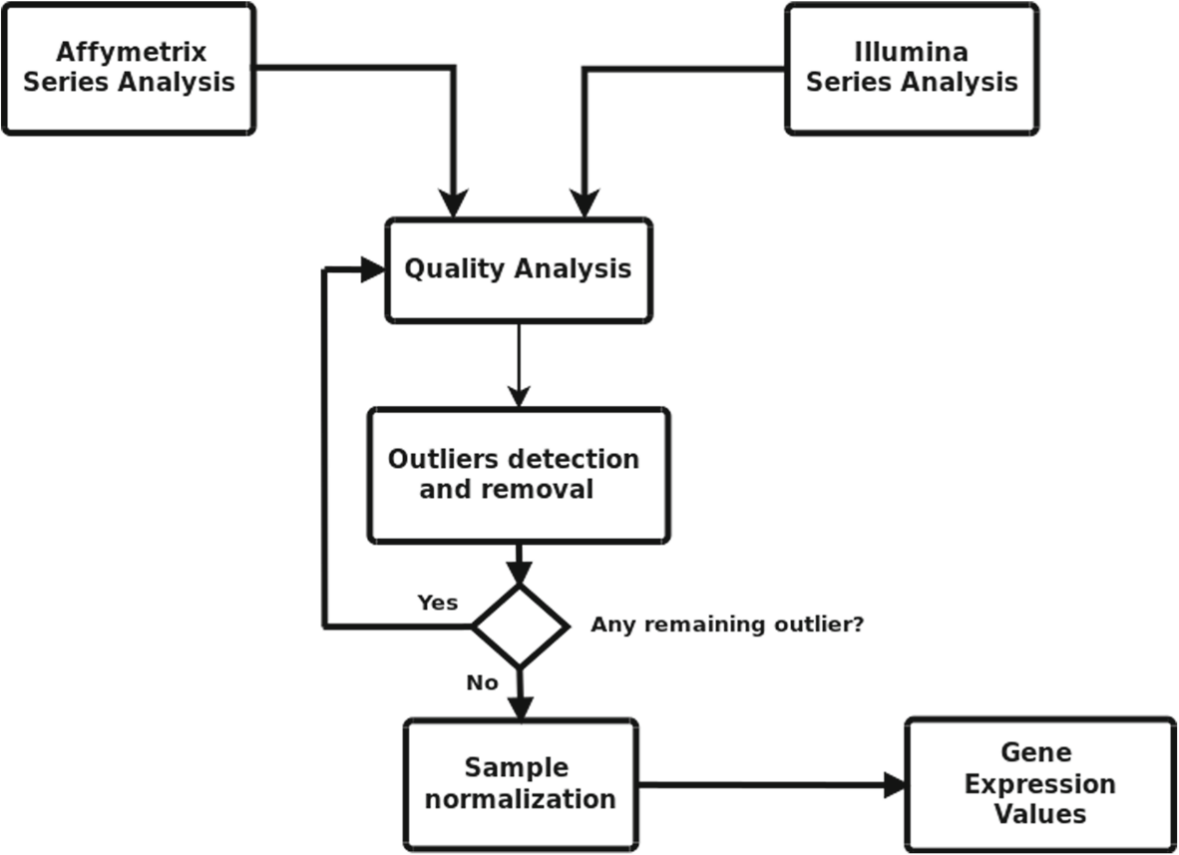
Microarray gene expression pipeline

The first step in the methodology for microarray data is to put together all the selected series, independently of their technology (Affimetrix or Illumina). Consequently, a quality analysis assessment was performed across the series, in order to detect and consequently remove any possible outlier. This outliers detection and removal was performed through arrayQualityMetrics R package [27], which computes the Kolmogorov-Smirnov statistic *K*_*a*_ between the distribution of each array and the distribution of the pooled data. Next, sample normalization was performed using the limma R package normalizedBetweenArrays function [10], in order to remove dynamic expression variability between samples. Once the samples were normalized, the expressed gene values were obtained. Figure 1 outlines the microarray data analysis pipeline.

### RNA-Seq pipeline

**Fig. 2:**
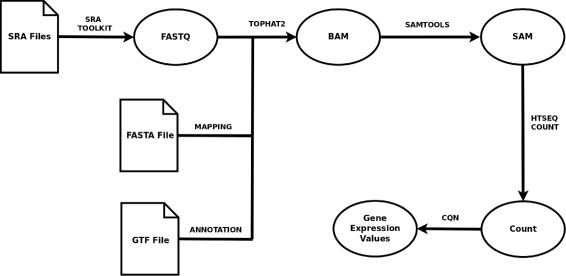
RNA-Seq gene expression integration pipeline

The pipeline proposed by Anders et al. [28] has been followed for the extraction of RNA-Seq data as it is shown in Fig. 2. Starting from the SRA original files, several tools like sra-toolkit [29], tophat2 [30], bowtie2 [31], samtools [32] and htseq [33] have been used to obtain the read count for each gene. Once the read count files were obtained, the expression values were calculated using the cqn and the NOISeq R packages [34].

### Integrated pipeline

**Fig. 3:**
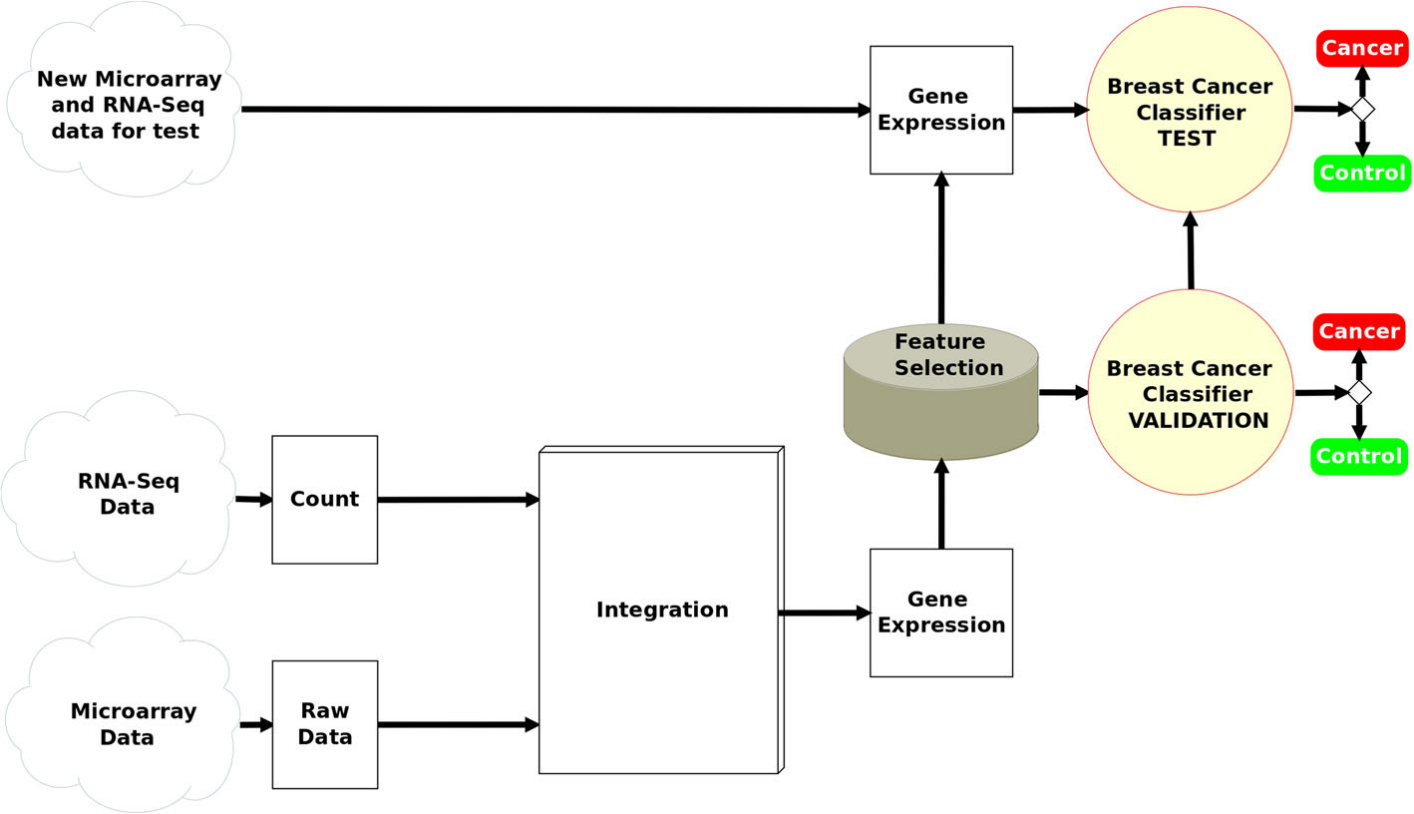
Integrated pipeline followed for this study

A new data processing pipeline is proposed in this work which extends the classical gene expression data analysis pipeline in two ways. On one hand, this pipeline integrates data from both microarray and RNA-Seq technologies. Furthermore, once the integration has been carried out, a gene selection process and an assessment through a classification process were performed, using separated training and test datasets. The workflow of the entire pipeline is shown in Fig. 3.

In a first step, sample integration of data from both microarrays and RNA-Seq technologies has been carried out using the merge function from base R package. Once the gene expression values have been obtained for each technology separately, a normalization of all joint technologies was applied using the normalizedBetweenArrays function cited before over all datasets available (see Table 1). These tasks are essential in order to have available a right normalization of the biological data and its subsequent processing [35, 36]. We have to note that each of the series in Table 1 were originally differently quantified depending on the respective technology and manufacturer.

The next steps in the pipeline for gene expression levels calculation and extraction of DEGs, were made only over the training dataset, thus leaving the test dataset for later assessment.

Gene extraction was performed at different levels using the limma R package, both at individual levels (microarray data and RNA-Seq data separately) and at integrated level (joined microarray and RNA-Seq data).

### Classification

Once a set of possible target genes which can be considered as biomarkers for breast cancer were identified, we proceeded to assess the results through three different classification technologies: SVM, RF and k-NN. The main objective of this stage is the validation of the behavior of the selected genes at the arrival of new unseen samples. The selected genes and the training dataset were used for designing the classification models, which were later evaluated over the test dataset.

- **SVM:** These models are supervised learning algorithms which assign categories to new samples. This algorithm is based on the idea of separating data from different categories through a hyperplane. The algorithm calculates the maximum-margin hyperplane that maximizes the distance between different classes. For overlapped data, this type of models turn a reduced space into a higher dimensional space using a kernel function, in order to perform the classification in this new space. Moreover, the algorithm tolerates making classification errors, which are controlled by the *γ* hyperparameter, in order to improve the generalization capability of the model [20, 21].
- **RF:** This method grows many single classification trees with the purpose of building a forest of classification trees. For the classification, the algorithm assigns the input vector to be classified to each tree of the forest. Once that each individual tree performs classification, the forest chooses the class having the largest number of votes over all the trees. After each tree is built, all of the data are run down the tree, and proximities are computed for each pair of cases. If two cases occupy the same terminal node, their proximity is increased by one. At the end of the run, the proximities are normalized by dividing by the number of trees. Proximities are used with the aim of replacing missing data, locating outliers and producing illuminating low-dimensional views of the data [22, 37].
- **k-NN:** This supervised method is based on assigning to a new unseen sample, the class corresponding to the predominant one in the k nearest neighbors (most similar samples) from the known labeled data. It is a well-known fast and easy-to-use technique which however provides a comparable performance to other well-known more complex techniques [23, 38].

Ten-fold cross-validation was used over the training dataset to obtain the optimal hyperparameters for these methodologies: *σ* (kernel width) and *γ* for SVMs, *number of trees* for RF and *k* for k-NN.

### Gene ranking: mRMR

Additionally, a feature selection process was performed through the mRMR [19] algorithm over the candidate biomarkers, with the objective of finding a reduced subset of genes that gives similar classification accuracy than the initial complete set of genes. In this way, the reduction of the number of genes allows the creation of a more simple and interpretable classifier, as well as more computationally efficient, while maintaining the robustness of the method. This algorithm creates a ranking of features, DEGs in our case. mRMR algorithm uses mutual information as the criterion for variables relevance, computing relevance and redundancy among variables (i.e. genes), and sorting them so that they bring largest relevance with respect to the class (cancer/no cancer) and, at the same time, they have lowest redundancy among themselves. Therefore, this algorithm will rank in first position the gene that contains the maximum relevance information, but the following genes will provide also minimum redundant information (apart from maximum relevance as regards to the class) with respect to the already selected genes, and so forth.

## Results and discussion

This section will focus on presenting and discussing the obtained results coming from the experimentation process followed in this study. It is divided into two subsections: first subsection shows the results for the process of obtaining the set of DEGs; while second subsection will show the results of the classification process making use of the former set of genes.

### Integrated gene expression

This subsection describes the process and results of the DEGs extraction. As it was previously stated in the methods section, series belonging to different technologies and platforms have been integrated. The objective of this integration is twofold: first, to increase the number of samples that will be used as input to our method, thus improving the robustness and stability of the results. Second, the obtained results will be independent of a single technology, as they proceed from different sources. The presence of RNA-Seq samples increases the dynamical midrange of the genes, making the results more accurate and robust. Furthermore, the number of available samples is greatly increased thanks to the availability of microarray data stored in public repositories.

**Fig. 4:**
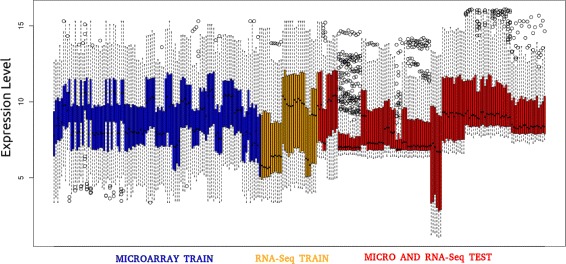
Expression profile of training and test datasets before normalization

**Fig. 5:**
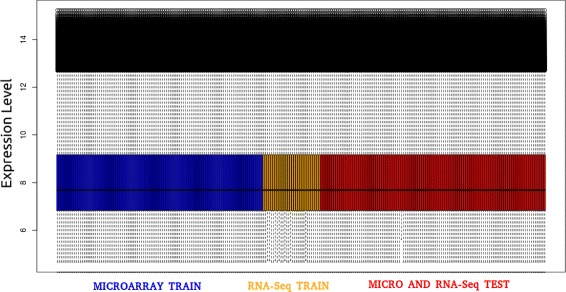
Expression profile of training and test datasets after normalization

When working with heterogeneous data, normalization is one of the most sensitive steps in the whole process, as a mistake in this step could cause interpretation errors, and could lead to a false set of expressed genes. Figure 4 shows the need of normalization for both training and test datasets together due to the difference of the dynamic range between samples. To this end, both training and test datasets have been subjected to a joint normalization using the normalizeBetweenArrays function from the limma R package, thus achieving the same dynamic range for all the samples. Figure 5 shows the results once the joint normalization was applied. As it can be seen, the dynamic range between samples has been corrected. In the next step, only the training dataset will be used in the process for identifying the DEGs.

**Fig. 6:**
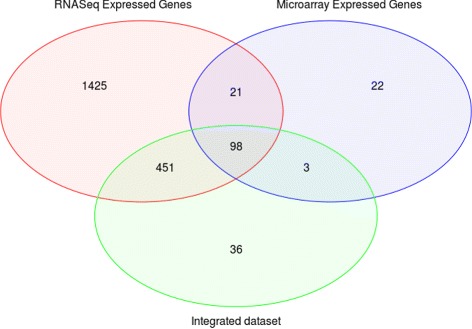
Intersection of expressed genes in RNA-Seq, microarray and the integrated dataset

We therefore proceeded to identify the DEGs both for each technology separately (microarray & RNA-Seq) and for the integrated dataset. Several restrictions were imposed in order to determine the expressed genes: the fold change in the expression values of the selected genes was set to be greater or equal than 2 and the *p*-value was set to be less or equal than 0.001. These constraints ensure that the chosen expressed genes are statistically significant, therefore showing different behavior between patient and healthy samples. These restrictions were applied to the three microarray, RNA-Seq and integrated datasets, so that three sets with different expressed genes were obtained. Finally, through the intersection of the three groups of expressed genes, a total of common 98 DEGs were found. These genes comply with the restrictions and they are differentially expressed in all datasets as the intersection shows (Fig. 6). Consequently, the obtained genes are differentially expressed independently of the gene expression technology, excluding possible noisy genes.

**Fig. 7:**
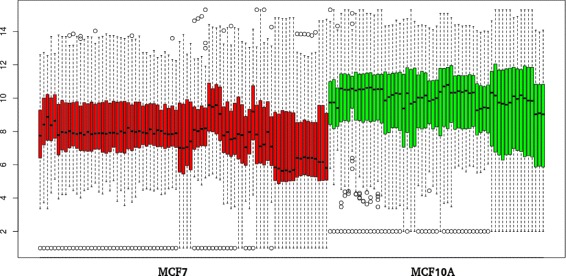
Gene expression values boxplot for the set of 98 expressed genes. Figure shows significant differences between expression values for MCF7 and HS578T cancer cell lines and MCF10A non-cancer cell line

A boxplot of the mean gene expression values of the 98 DEGs for the samples in the training dataset is shown in Fig. 7. It shows a clear differentiation between the average value of the cancer cell lines samples and the average value of the MCF10A non-cancer cell line samples. Furthermore, the statistical information of the intersection set of 98 DEGs is shown in Table 2.

**Table 2:** List of 98 expressed genes obtained with limma as the intersection of microarray, RNA-Seq and integrated dataset

Genes names	∣*l* *o* *g* *F* *C*∣≥2	t	p-val	adj.p.val	B
KRT19	7.993	11.072	8.124E-21	2.449E-19	36.607
KRT6A	-7.800	-13.558	3.347E-27	2.503E-25	51.214
NNMT	-7.584	-11.544	4.951E-22	1.780E-20	39.384
VIM	-7.261	-15.117	3.917E-31	5.046E-29	60.213
AKR1B1	-6.943	-11.437	9.357E-22	3.265E-20	38.753
SFRP1	-6.866	-18.820	4.925E-40	1.904E-37	80.570
TGFBI	-6.701	-14.299	4.424E-29	4.174E-27	55.515
MT1E	-6.650	-15.281	1.537E-31	2.079E-29	61.142
C3	-6.569	-15.928	3.857E-33	6.589E-31	64.805
BMP7	6.406	13.058	6.330E-26	3.910E-24	48.292
KRT5	-6.229	-9.125	7.460E-16	1.062E-14	25.273
CXCL1	-6.145	-13.526	4.030E-27	2.986E-25	51.030
S100A2	-6.016	-9.582	5.249E-17	9.014E-16	27.902
KRT7	-5.991	-11.975	3.850E-23	1.643E-21	41.922
TNS4	-5.866	-25.125	1.651E-53	3.829E-50	111.284
EEF1A2	5.764	8.956	1.979E-15	2.656E-14	24.307
CLMP	-5.631	-11.238	3.037E-21	9.781E-20	37.583
IFI16	-5.543	-9.230	4.073E-16	6.036E-15	25.872
LAMC2	-5.426	-12.346	4.247E-24	2.015E-22	44.112
IGFBP4	5.412	13.779	9.173E-28	7.406E-26	52.501
FAM83A	-5.328	-14.042	1.974E-28	1.741E-26	54.028
SYTL2	5.283	11.883	6.617E-23	2.725E-21	41.384
SNAI2	-5.169	-9.731	2.204E-17	4.010E-16	28.762
DNER	-5.152	-11.859	7.620E-23	3.114E-21	41.244
PRKCDBP	-5.105	-10.241	1.105E-18	2.434E-17	31.730
ALOX15B	-5.088	-16.524	1.353E-34	2.896E-32	68.133
IGFBP5	5.085	8.165	1.755E-13	1.735E-12	19.871
BNC1	-5.072	-16.335	3.889E-34	7.697E-32	67.085
GFRA1	5.021	6.872	1.958E-10	1.223E-09	12.955
DSC3	-4.999	-17.145	4.296E-36	1.181E-33	71.561
PTGES	-4.990	-17.489	6.479E-37	1.947E-34	73.440
TFF1	4.925	4.857	3.168E-06	1.023E-05	3.497
RAB25	4.864	8.521	2.368E-14	2.683E-13	21.851
KRT14	-4.863	-6.445	1.768E-09	9.652E-09	10.794
EFEMP1	-4.855	-10.020	4.059E-18	8.275E-17	30.440
SLPI	-4.793	-10.194	1.455E-18	3.128E-17	31.457
SDPR	-4.728	-12.002	3.264E-23	1.401E-21	42.086
FBP1	4.707	6.789	3.017E-10	1.848E-09	12.530
EPCAM	4.662	8.150	1.906E-13	1.878E-12	19.790
GNA15	-4.570	-15.676	1.614E-32	2.495E-30	63.382
HTRA1	-4.527	-10.906	2.178E-20	6.152E-19	35.627
RAC2	-4.524	-11.727	1.669E-22	6.433E-21	40.465
CLCA2	-4.411	-9.272	3.189E-16	4.828E-15	26.115
GPX1	-4.384	-6.773	3.281E-10	1.994E-09	12.448
EMP3	-4.383	-9.299	2.728E-16	4.176E-15	26.269
SERPINB5	-4.371	-8.314	7.600E-14	8.016E-13	20.698
TSPYL5	4.317	6.297	3.735E-09	1.943E-08	10.062
GSTP1	-4.242	-5.846	3.433E-08	1.523E-07	7.892
SLC2A10	4.216	11.411	1.088E-21	3.782E-20	38.602
LDHB	-4.182	-5.892	2.745E-08	1.238E-07	8.111
VSTM2L	-4.146	-11.277	2.409E-21	7.852E-20	37.813
BIRC3	-4.079	-13.064	6.110E-26	3.799E-24	48.327
ABLIM3	-4.000	-12.337	4.481E-24	2.113E-22	44.059
TFCP2L1	-3.874	-11.847	8.202E-23	3.344E-21	41.171
DSG3	-3.820	-8.387	5.035E-14	5.469E-13	21.105
SLC26A2	-3.798	-13.491	4.947E-27	3.632E-25	50.826
C3orf14	3.763	7.772	1.558E-12	1.358E-11	17.715
IL20RB	-3.667	-8.868	3.262E-15	4.229E-14	23.812
FXYD5	-3.623	-5.585	1.191E-07	4.882E-07	6.679
GSTM3	3.590	9.622	4.161E-17	7.268E-16	28.133
ADRB2	-3.572	-9.968	5.512E-18	1.099E-16	30.136
EMP1	-3.535	-7.622	3.543E-12	2.907E-11	16.905
IGFBP7	-3.530	-4.676	6.866E-06	2.104E-05	2.751
GJB5	-3.517	-12.456	2.225E-24	1.097E-22	44.755
HENMT1	3.514	7.953	5.732E-13	5.316E-12	18.702
ZBED2	-3.507	-6.452	1.705E-09	9.338E-09	10.830
MSLN	-3.504	-8.558	1.917E-14	2.217E-13	22.061
IL18	-3.415	-9.270	3.223E-16	4.864E-15	26.104
TRIM29	-3.395	-9.588	5.081E-17	8.735E-16	27.934
OSR2	3.346	8.380	5.238E-14	5.671E-13	21.066
LAMB1	-3.346	-6.972	1.162E-10	7.510E-10	13.468
UCP2	3.332	5.788	4.539E-08	1.979E-07	7.620
CPVL	-3.331	-7.870	9.043E-13	8.152E-12	18.253
KRT81	-3.320	-5.133	9.424E-07	3.334E-06	4.670
S100A8	-3.292	-5.698	6.982E-08	2.957E-07	7.200
TP53I3	-3.242	-11.149	5.160E-21	1.589E-19	37.057
FOXA1	3.226	5.576	1.241E-07	5.069E-07	6.640
SLC24A3	3.211	6.190	6.356E-09	3.184E-08	9.541
PNLIPRP3	-3.200	-7.998	4.470E-13	4.207E-12	18.948
INHBB	3.180	7.756	1.698E-12	1.468E-11	17.630
RAB38	-3.129	-9.539	6.781E-17	1.137E-15	27.649
ZBTB16	-3.112	-8.869	3.251E-15	4.217E-14	23.816
PLD5	-3.070	-11.039	9.925E-21	2.960E-19	36.408
DFNA5	-3.047	-7.565	4.835E-12	3.890E-11	16.599
FKBP5	-2.988	-10.435	3.528E-19	8.458E-18	32.863
CD109	-2.986	-7.196	3.541E-11	2.475E-10	14.637
CASP1	-2.955	-6.388	2.367E-09	1.267E-08	10.509
SULT1E1	-2.903	-7.749	1.763E-12	1.513E-11	17.594
FAM174B	2.779	5.557	1.353E-07	5.493E-07	6.555
PDZK1IP1	-2.752	-7.028	8.611E-11	5.667E-10	13.743
TNNI2	-2.750	-7.896	7.842E-13	7.133E-12	18.393
CAV1	-2.727	-5.028	1.503E-06	5.131E-06	4.217
IRX4	-2.714	-7.628	3.433E-12	2.825E-11	16.936
KRT80	2.706	5.268	5.131E-07	1.895E-06	5.259
FOXO1	-2.649	-8.921	2.408E-15	3.188E-14	24.113
SNCA	-2.635	-8.533	2.211E-14	2.526E-13	21.919
TBL1X	2.565	9.676	3.043E-17	5.434E-16	28.442

Table 2 shows five statistics values computed by the limma package (logFC, t-statistic, *p*-value, adj.p.val. and B). The log-fold change (*logFC*) represents the difference between breast cancer and control expressed values. If ∣*l* *o* *g* *F* *C*∣≥2 it means that there exists significant differences between cancer and control values. The second value in Table 2 is the moderated t-statistic, which is the ratio between the log2-fold change value for each gene and it respective standard error. The next value is the *p*-value (*p*-val) which represents the probability of obtaining a result equal or higher than what it was observed when the null hypothesis is true. The adjusted *p*-value indicates which proportion of comparisons within a family of comparisons (hypothesis tests) are significantly different. The B-statistic (*B*) is the log-odds that a given gene is differentially expressed.

**Fig. 8:**
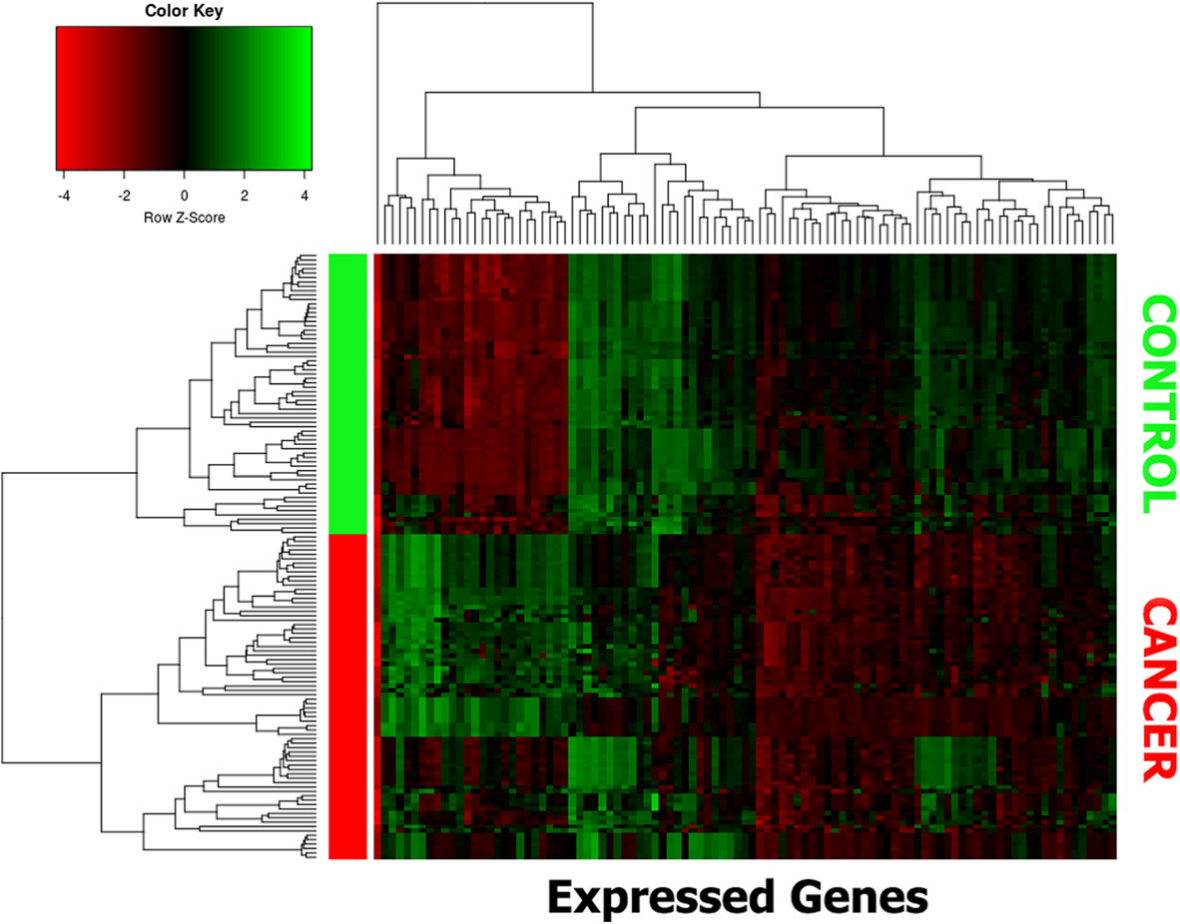
Hierarchical cluster using the 98 invariant expressed genes

Figure 8 depicts a hierarchical clustering using the list of 98 invariant expressed genes. As it can be seen, the cluster is split into two group of samples, one belonging to control samples and the other to breast cancer samples. Thus verifying that the obtained genes are robust and clearly differentiating.

#### Classification results

Once the DEGs were identified in the previous subsection, this subsection assesses the performance of these genes through a classification process when new samples are presented. For that purpose, the classification algorithms SVM, RF and k-NN have been implemented. The whole training dataset formed by 132 samples has been used as the input data for the classifier (Table 1). The 98 DEGs values were normalized to range between [-1,1], and have been chosen as classification features, ordered by a mutual information-based ranking provided by the mRMR algorithm. Moreover, for a further assessment of the classifier against new unseen samples, a test dataset made up of 126 samples has been equally normalized and used for testing (Table 1).

Following the proposed integrated pipeline in this work (see Fig. 3), once the samples were correctly integrated and the 98 DEGs were found, a classification method using these genes has been applied. Results for all the algorithms in the validation stage using the 98 genes reached an accuracy equal to 100%. Therefore, all samples belonging to the training dataset were successfully classified. When the classifier using 98 genes was applied to test samples, an accuracy above 95% was reached by the three algorithms, rising up to a 97% in the case of SVMs and RFs, thus confirming the robustness of the proposed pipeline approach (see Table 3).

**Table 3:** Training and test classification accuracies for SVMs, RFs and k-NN algorithms

	1 Gene	6 Genes	98 Genes
Training accuracy			
Support vector machines	98.5%	100%	100%
Random forest	97.8%	99.2%	100%
k-Nearest neighbor	98.5%	99.2%	100%
Test accuracy			
Support vector machines	86.5%	96.8%	97.6%
Random forest	82.3%	87.4%	97.4%
k-Nearest neighbor	84.4%	94.1%	94.9%

**Fig. 9:**
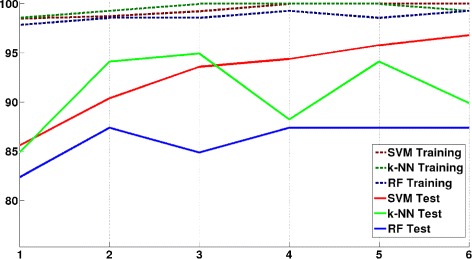
Validation and test classification results with SVM, RF and k-NN using the most relevant genes obtained by mRMR

Afterwards, a feature selection process has been applied in order to reduce the cardinality of the 98 DEGs. As a result, the mRMR algorithm returned a gene ranking based on mutual information. Figure 9 shows the validation (10-CV over the training dataset) and test results using the three algorithms: SVMs, RFs and k-NN. These validation results are above 98% using only the first gene of the ranking for classification for the three algorithms, and above 99.2% using a reduced set of the first six genes in the ranking. Moreover, classification results when using the new 126 unseen samples of the test set and the three methods, rose up to coherent results with an accuracy of 96.8% using SVMs, 94.1% using k-NN, nevertheless lower for RFs with a 87.4%. Therefore, the classifier performs in a similar way to the behavior observed in the validation results for two of the classifiers. Consequently, the main set of 98 DEGs was reduced to the later six genes set, which allow discerning if new samples are cancerous or not, with an expected error around a 3.2% when using a SVM classifier.

These differences in performance among classification techniques are usual in this type of problems, and a number of papers comparing classification techniques for biological data can be found in the literature [37, 39–41]. In the results above-mentioned, using only 6 genes, SVMs attains an optimal performance near that attained using the complete set of 98 genes. This behavior is also seen in the k-NN technique, although with a lower performance. RF on the other hand obtains similar results than SVMs when the complete set of 98 genes are used, but fails to design a simpler classifier with a low number of genes with optimal performance [39, 40]. Thus, these results support the design of an optimal classifier based on SVMs with only six genes attaining the excellent aforementioned results.

Finally, once the potential biomarker genes were identified as the reduced subset of six genes, a literature review and biological study was done in order to reveal the relation between those genes and their involvement in breast cancer (Table 4). The first five of these six genes have been formerly reported as genes involved in breast cancer, whilst the sixth gene is present in breast cancerous tissue, although with no evidence of a direct implication with breast cancer development. This means that the results attained by the proposed integrated pipeline are coherent, as the reduced subset of six genes is formed by genes related with breast cancer. Furthermore, these genes can be used for classification and diagnosis purposes over new unseen samples. They can be designated as a new breast cancer biomarker signature when these types of cell lines data are present.

**Table 4:** Relationship of the top 6 expressed genes with breast cancer

Gene symbol	Gene name	Relationship between protein and breast cancer
SFRP1	Secreted frizzled-related protein 1	Inhibition of SFRP1 increases the proliferation, migration and invasion of breast cancer cells. SFRP1 exerted this function by activating Wnt/ *β*-catenin signaling pathway in breast carcinogenesis [42, 43].
GSTM3	Glutathione S-transferase mu 3	GSTM3 is suggested as an important modifier that impacts on individual susceptibility to develop breast cancer among premenopausal women [44]. High expression of GSTM3 is related to protective genotypes against breast cancer
SULT1E1	Gulfotransferase family 1E member 1	SULT1E1 is an enzyme that catalyzes the sulfation of active 17 *β*-estradiol into inactive form. SULT1E1 is highly expressed in normal mammary epithelial cells and rarely expressed in breast cancer cells. However, its over-expression in breast carcinomas is considered to retard tumor cell growth by arresting cell cycles and inducing apoptosis and may thus improve the prognosis of breast cancer [45, 46].
MB	Myoglobin	MB plays a functional role in breast cancer progression by promoting the growth of fully oxygenated cells through the control of fatty acid homeostasis and lipogenesis [47, 48]. MB is dose-dependent downregulated by 17 *β*-estradiol in breast cancer cells [49].
TRIM29	Tripartite motif containing 29	TRIM29 is considered a breast cancer tumor suppressor. Low TRIM29 expression in breast cancer is associated with more aggressive tumor features. Suppression of the oncogenic transcription factor TWIST1 expression is one mechanism suggested by which TRIM29 functions as a suppressor of breast cancer development [50].
VSTM2L	V-set and transmembrane domain containing 2 like	Although VSTM2L is detected in breast cancer tissues, to date there are no relation between its expression and breast cancer development in the current literature.

**Fig. 10:**
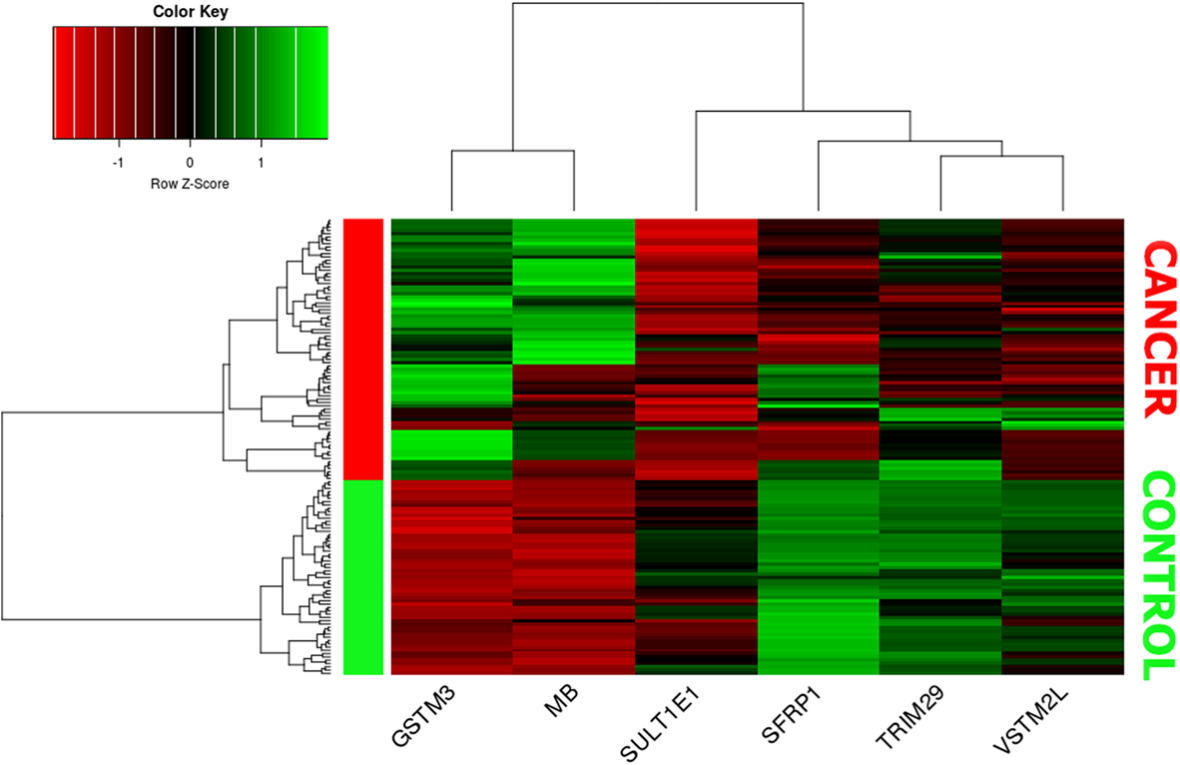
Hierarchical cluster over healthy and breast cancer samples using the top 6 genes

Figure 10 shows a hierarchical cluster built with the small six genes subset. Two distinct groups are clearly identified, as it also happened in Fig. 8: one matching control samples and the other matching breast cancer samples. Therefore, this indicates that the expression profiles of these genes constitute a possible diagnosis criteria for breast cancer.

**Fig. 11:**
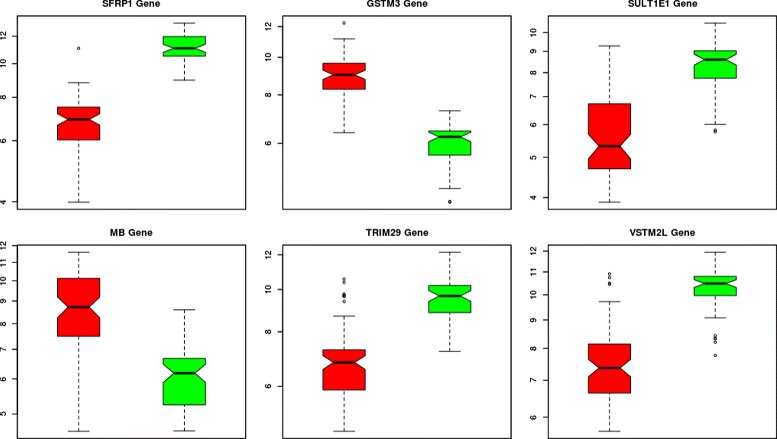
Average expression value boxplots of the six most relevant genes obtained in this study

Figure 11 shows a boxplot for each of the six genes representing the average expression value for the cancerous samples (red), and control samples (green). As it can be seen, average expression values between cancerous and control samples are clearly differentiated, thus reaffirming their potential as breast cancer biomarkers.

## Conclusions

This work has presented the possibility of integrating data from different gene expression analysis technologies. On the one hand, microarrays, which have been widely used in the last two decades and, on the other hand, RNA-Seq that is the technology meant to replace microarrays definitely.

An exhaustive search from the NCBI-GEO public repository has been performed in order to collect breast cancer samples from both technologies. The intersection of DEGs in microarray, RNA-Seq, and the integrated dataset, has allowed identifying a set of candidates biomarkers for diagnosis of this disease.

Thereafter, feature selection through mRMR was applied in order to select the most relevant biomarkers subset. Three different classification models (SVMs, RFs, and k-NN) were designed from the training dataset and the selected DEGs and compared. These classifier were validated with the test dataset achieving outstanding results for the three algorithms when the complete set of 98 DEGs were used.

In conclusion, results show that the expressed genes can be designated as robust biomarkers for breast cancer diagnosis when specific cell lines samples are used. Furthermore, even with a small subset of six of those genes, a great validation accuracy was reached (99%). Also, classification results over new unseen data show great accuracy, specially over SVM classification (96.8%). Five of these top six genes have been formerly reported as genes that show biological relation with breast cancer, which reinforce the designation of the expression profiles of these genes for breast cancer diagnosis.

## Abbreviations

DEGs: Differentially expressed genes
k-NN: k-nearest neighbor
mRMR: Minimum-redundancy maximum-relevance
RF: Random forest
SVM: Support vector machines
